# Supplementary material for: Chromothripsis during telomere crisis is independent of NHEJ, and consistent with a replicative origin
Source: Genome Res. 2019 May;29(5):737–49. doi: 10.1101/gr.240705.118 (PMC6499312; doi:10.1101/gr.240705.118)
Supplement: Supplemental Material [file supp_gr.240705.118_Supplemental_file_1.zip › contigs/annotated_contigs/DB54/contig.2.DB54_length_904_mean_cov_11.5077433628.docx]

**DB54_length_904_mean_cov_11.5077433628**

```
CTGGGA|TAAGAGGCATGTGCCACCATGCCCAGCTGTTTTTTGTATTTTTAGTAGAGACGGGGTTTCACCATGTTGGCCAGGCTGGTCT
       >chr7:5512726-5513273 - E=4e-302
CGAACTCCTGACCTCAGGTGATCCACCCACCTTGGCCTCCCAAAGTGCTGGGATTACAGGCATGAGCCACCGCACCCGGCACGTGTGCT

CTTGAGGCTGGTGTATTTGCAGTGTCAGTTTGGGGCAGAGACCTCCTTTACCTGTCCTGGGCTCTGGGAAGAGGCTCGGCCCAAATGTG

GCAGGCGGGGGGAGGAGGCACCAGGATCCGGATGGGGACAGGGCTGCCGGCAGAGGGTGGGCTCCAGGGCTGCTGCAGAACGGAGCGGC

CAGGGTGGGGCCCCTCACATGAGCCAGGGACTCAGCAATGCCTCCTTCTGGCTTGGTTCTTGAGGTGATGGTGTTTCTCTGGCAGAAAT

GGCGACATAGGGCCAGGAGCCTCCCATCCTGCCCAACATTGTCTGAGCCGTTGCCTGCAGCTTGACATGTACACACCGTCCCCTGGTGA

TGTCTTGTGTCTGGCTGGA|G|AGTCTGGAGAACCGTGGCCTGGCTCCCTCGACCCGCATCTCACCTGGGACCCCAGAGATGCCCCCGG
                    >chr7:5647568-5647920 - E=8e-200
CCCCTGGCCTGGCTTCCCCCCAGAGGGCAGGGCCTGGGCAGGTCCCTCGCCCCGGGTTATTCCCCCGTCCCTTTCAGTTGGGCAGCTCC

TACCGATTCCTCCCGAGAGATCTCGTGAACGGCCCTTCCAGCCTCCCCCATCGGACTATGCTGCTCATTGGCTGCAGCCTCGGGAATCT

CGGCCACGTGGTAGGCGCCAAATTTTGCAGAATGAATGGAATTCGGAATCAGCTGGTGGCTCCGGGGACCTTAGGGTCATTCTAGGTCA

GCTCCATGGGTGGAAAT
```
